# High Prevalence of tuberculosis infection among medical students in Makerere University, Kampala: results of a cross sectional study

**DOI:** 10.1186/0778-7367-71-7

**Published:** 2013-04-20

**Authors:** Henry Mugerwa, Denis K Byarugaba, Simon Mpooya, Penelope Miremba, Joan N Kalyango, Charles Karamagi, Achilles Katamba

**Affiliations:** 1 Clinical Epidemiology Unit, Department of Medicine, College of Health Sciences, Makerere University, P.O box 7072, Kampala, Uganda; 2 School of Veterinary Medicine, Makerere University, P.O box 7062, Kampala, Uganda; 3 Department of Pharmacy, College of Health Sciences, Makerere University, P.O box 7072, Kampala, Uganda

**Keywords:** Tuberculosis infection, Medical students, Veterinary students, Tuberculin skin test, Uganda, Prevalence

## Abstract

**Background:**

Uganda’s Ministry of Health registered a 12% increase in new Tuberculosis (TB) cases between 2001 and 2005. Of these, 20% were from Kampala district and most from Mulago national referral hospital where the largest and the oldest medical school is found. Medical students are likely to have an increased exposure to TB infection due to their training in hospitals compared to other university students. The study compared the prevalence of TB infection and associated factors among undergraduate medical and veterinary students in Makerere University, Uganda.

**Methods:**

This was a cross-sectional study with 232 medical and 250 veterinary undergraduate students. Socio-demographic and past medical history data was collected using questionnaires. A tuberculin skin test was performed on the volar aspect of the left forearm. An induration ≥10 mm in diameter after 48-72 hrs was considered positive. Logistic regression was used to determine association of independent variables with TB infection.

**Results:**

The prevalence of TB infection was higher in medical students (44.8%, 95% C.I= 38.4-51.3%) compared to veterinary students (35.2%, 95% C.I = 29.3-41.1%). The significant predictors of TB infection were: being a medical student (aOR=1.56, 95% CI = 1.05-2.31), male sex (aOR=1.75, 95% CI = 1.17-2.63), history of contact with a confirmed TB case (aOR=1.57, 95% CI = 1.06-2.31) and residing at home (aOR=2.08, 95% CI = 1.20-3.61). Among the medical students, having gone to a day compared to boarding high school (aOR=2.31, 95% CI = 1.06-5.04), involvement in extracurricular clinical exposure (aOR=3.39 95% CI = 1.60-7.16), male sex, residence at home, and history of contact with a TB case predicted TB infection.

**Conclusion:**

Medical students have a higher prevalence of TB infection than veterinary students probably due to increased exposure during training. There is a need to emphasize TB infection control measures in hospitals and the general community.

## Background

One third of the world’s population is estimated to be infected with the latent form of tuberculosis (TB) [1]. In 2008, World Health Organization (WHO) estimated 9.4 million incident cases, 11.1 million prevalent cases and 1.8 million deaths from TB worldwide. Uganda is on the list of 22 high-burden TB countries in the world and in 2011, WHO estimated the prevalence of TB in Uganda to be at 193 per 100000 populations [2]. However an earlier study conducted between 2001 and 2002, estimated the prevalence of all forms of TB in Kampala to be 1400 per 100000 population [3]. The Ministry of Health registered a 12% increase in new TB cases between 2001 and 2005 of which 20% were from Kampala district and most of the cases were from Mulago national referral hospital [4] where the largest and the oldest medical school in Uganda is found.

Several studies have suggested a high risk of infection by *Mycobactrium Tuberculosis (MTB)* among medical students due to exposure to patients during training. A study conducted in Kampala among health care workers and students in 2005 found that 40% of medical students had latent tuberculosis infection (LTBI) [5-7]. A more recent study by Joseph K Lou among 288 undergraduate medical students in Makerere University College of Health Sciences (Medical school) estimated the prevalence of LTBI at 45.1% (unpublished dissertation, 2009). However, due to lack of a comparison group it was not possible to determine if the prevalence of TB infection in medical students was higher than that of other Makerere University students. There are currently no proper preventive measures for nosocomial TB infection for both the staff and the medical students on the wards and other hospital departments putting them at an increased risk of infection. Makerere University medical school uses the problem based learning approach. Students are exposed to clinical proceedings early on in their course of study. Many of them are less likely to be aware or have grasped the risk reduction measures when interfacing with patients. Because of this, there is a high likelihood of exposure to infectious diseases from patients. The ripple effect of this may have a multiplier effect when these students in turn become the source of infection to the patients, fellow students and the community at large.

Students in Makerere University School of Veterinary Medicine (Veterinary school) are closely similar to those in the medical school in regard to their background and the years they spend in training at the University apart from the risk of exposure to patients. They were therefore considered to be a suitable comparison group. Given that Uganda is a high-burden TB country, there is already a high risk for transmission of TB to the students from communities where the disease is rampant. TB infection acts as a reservoir for future disease and infections. Among those infected, 10% will develop active TB disease in their lifetime [8].

The study aimed at comparing the prevalence and associated factors of TB infection among undergraduate medical and veterinary students in Makerere University.

## Methods

A cross-sectional study was conducted between February and March 2011 among undergraduate medical and veterinary students in Makerere University.

Makerere University is located in the northern outskirts of Kampala, the capital city of Uganda. It has about 30,000 undergraduate students. The medical school is located within Mulago hospital complex - a national referral hospital. It has a total of 985 undergraduate students pursuing bachelor’s degrees in Medicine and Surgery, Dental Surgery, Nursing, Pharmacy and Medical Radiology. The veterinary school is located at Makerere University main campus, a kilometre away from Mulago hospital. It has a total of 492 undergraduate students pursuing bachelor’s degrees in Veterinary Medicine, Biomedical Laboratory Technology, Animal Production and Technology and Wildlife Health and Management. We enrolled students in medical school pursuing medicine and surgery, dental surgery and nursing from a total of 787 students. We enrolled students from any of the four courses offered at veterinary school.

We used the formula for comparison of two proportions [9] and estimated a sample size of 278 students from each school as sufficient for the comparison of prevalence of TB infection. This was based on assumptions of 45% prevalence of TB among medical students, 30% difference in TB infection among medical and veterinary students indicating a clinically significant difference since we did not have prior data on prevalence of TB infection in veterinary students, 90% power, and 5% level of significance. The students were sampled from the respective schools using simple random sampling method. Class registers were used to assign numbers to all the students, and computer generated random numbers were used to randomly select those that were included in the study.

Self administered questionnaires were used to collect data on socio-demographics, residence, course and year of study, Bacille Calmette-Guerin (BCG) vaccination status, exposure to TB case, use of respiratory protective masks, chronic co-morbidities, smoking history, alcohol consumption, knowledge about TB transmission, engagement in extra clinical activities outside normal study periods, TB symptoms and past medical history. The participants were examined for BCG vaccination scar. TB infection was determined using an intradermal administration of 0.1 ml of a 2 tuberculin units purified protein derivative (PPD) (TUBERCULIN PPD RT 23, STATENS SERUM INSTITUTE, Copenhagen, Denmark), given on the volar aspect of the left forearm using a 29 gauge needle by trained clinical officers. The transverse diameter of the induration was measured 48-72 hours later using a transparent ruler. A Tuberculin Skin Test (TST) that produced an induration of ≥ 10 mm was considered positive. The collected data was cleaned and entered into EpiData version 3.1 software (EpiData association, Odense, Denmark) with checks to minimize errors. It was then exported to STATA version 10.0 (STATA Corp, College Station, TX, USA) software for analysis.

Continuous variables were compared using t-tests or Mann-Whitney U test as appropriate while categorical variables were compared using chi-squared or fisher’s exact tests. The chi-squared trend test was used to compare proportions of TB infection for the ordinal independent variable (year of study). Odds ratios (OR) and their 95% confidence intervals (CI) were estimated to measure association of independent variables with TB infection at bivariate and multivariate analysis using logistic regression.

The study was approved by Makerere University College of Health Sciences Research and Ethics Committee and Makerere University School of Veterinary Medicine Research and higher degrees committee. Informed written consent was obtained from all study participants. Students who had a positive TST result and a suggestive history for TB were further evaluated for possible active TB disease by a chest x-ray plus sputum examination.

## Results

We enrolled 232 undergraduate medical students and 250 undergraduate veterinary students into the study. The veterinary students were older, and had higher proportions of males, students residing at home or in private halls, and those engaging in extracurricular clinical exposure. On the other hand, medical students had a higher proportion of students exposed to a TB case and in the clinical years of study (Table 1).

**Table 1:** Socio-demographic characteristics of undergraduate veterinary and medical students in Makerere University, Kampala, Uganda 2011

**Characteristics**	**Veterinary school (N=250) n (%)**	**Medical school (N=232) n (%)**	**p-value**
**Mean age ±SD (years)**	23.04 (±3.38)	22.41 (± 2.49)	0.021
**Sex (male)**	171 (68.4)	137 (59.1)	0.033
**High school (boarding school)**	206 (83.1)	186 (82.3)	0.826
**Nationality (Ugandan)**	243 (97.2)	222 (95.7)	0.369
**Residence**			
University hall	74 (29.6)	164 (70.7)	<0.001
Private hall	80 (32.0)	36 (15.5)	<0.001
Renting a room	52 (20.8)	14 (6.0)	<0.001
Home	44 (17.6)	18 (7.8)	0.001
**Extra clinical exposure**	78 (31.7)	43 (18.5)	0.001
**Contact with a tuberculosis case**	90 (36.0)	121 (52.2)	<0.001
**Clinical year**	53 (21.2)	91 (39.2)	<0.001
*BCG^**† **^**vaccination scar present**	150 (62.8)	170 (75.9)	0.002
**History of smoking**	12 (4.8)	9 (3.9)	0.621
**History of alcohol consumption**	100 (40.0)	82 (35.3)	0.292
*Knowledge that tuberculosis is commonly transmitted by cough	130 (54.9)	126 (58.9)	0.389

*some data is missing – some participants did not give a response.

^†^Bacille Calmette-Guerin.

The prevalence of TB infection was higher among medical students (44.8%, 95% C.I 38.4 – 51.3) than veterinary students (35.2%, 95% C.I 29.3 – 41.1). Although there appeared to be a trend of increasing TB infection with the year of study among the medical students especially in the first four years, it was not statistically significant (p=0.057). There was no apparent trend of TB infection among the veterinary students (p=.384) (Tables 2 and 3).

**Table 2:** Prevalence of tuberculosis infection among 232 undergraduate medical students in Makerere University, Kampala, Uganda 2011

**Year of study**	**Number tested**	**Number with positive tuberculin skin test**	**Prevalence of positives (95% C.I)**
1	41	15	36.6 (21.2-52.0)
2	56	22	39.3 (26.1-52.5)
3	44	21	47.7 (32.4-63.1)
4	73	37	50.7 (38.9-62.4)
5	18	9	50.0 (24.4-75.6)
Total	232	104	44.8 (38.4-51.3)

**Table 3:** Prevalence of tuberculosis infection among 250 undergraduate veterinary students in Makerere University, Kampala, Uganda 2011

**Year of study**	**Number tested**	**Number with positive tuberculin skin test**	**Prevalence of positives (95% C.I)**
1	61	19	31.1 (19.2 - 43.1)
2	80	28	35.0 (24.3 - 45.7)
3	56	21	37.5 (24.4 - 50.6)
4	32	11	34.4 (17.0 - 51.8)
5	21	9	42.9 (19.8 - 65.9)
Total	250	88	35.2 (29.3 – 41.1)

When students from both schools were considered together in multivariate logistic analysis, being a medical student (aOR = 1.56, 95% C.I 1.05-2.31), male sex (aOR = 1.75, 95% C.I 1.17-2.63), residence at home (aOR = 2.08, 95% C.I 1.20–3.61) and a history of contact with a TB case (aOR = 1.57, 95% C.I 1.06-2.31), were associated with TB infection (Table 4).

**Table 4:** Predictors of tuberculosis infection among undergraduate medical and veterinary students, Makerere University, Kampala, Uganda, 2011

**Characteristic**	**Bivariate analysis**		**Multivariate analysis**	
	**Unadjusted OR (95% C.I)**	**p-value**	**Adjusted OR (95% C.I)**	**p-value**
**Male gender**	1.54 (1.05-2.27)	0.029	1.75 (1.17-2.63)	0.007
**Residence (home)**	1.84 (1.05-3.23)	0.034	2.08 (1.20-3.61)	0.009
**School (Medical)**	1.50 (1.04-2.16)	0.031	1.56 (1.05-2.31)	0.027
**Clinical year (4&5)**	1.42 (0.96-2.11)	0.08	1.17 (0.77-1.78)	0.455
**Contact with a Tuberculosis case**	1.58 (1.09-2.28)	0.015	1.57 (1.06-2.31)	0.024

OR = Odds Ratio.

When medical students were analysed separately, male sex (aOR = 2.12, 95% C.I 1.15-3.93), having gone to a day compared to a boarding high school (aOR = 2.31, 95% C.I 1.06-5.04), residence at home (aOR = 5.27, 95% C.I 1.51-18.39), history of contact with a TB case (aOR = 1.91, 95% C.I 1.01-3.61) as well as involvement in extracurricular clinical exposure (aOR=3.39, 95% C.I 1.60-7.16) were associated with TB infection (Table 5).

**Table 5:** Predictors of tuberculosis infection among undergraduate medical students in Makerere University, Kampala, Uganda, 2011

**Characteristic**	**Bivariate analysis**		**Multivariate analysis**	
	**Unadjusted OR (95% C.I)**	**p-value**	**Adjusted OR (95% C.I)**	**p-value**
**Male gender**	2.02 (1.18-3.47)	0.011	2.12 (1.15-3.93)	0.017
**High school (day school)**	2.75 (1.35-5.61)	0.005	2.31 (1.06-5.04)	0.034
**Residence (home)**	5.20 (1.64-16.48)	0.005	5.27 (1.51-18.39)	0.009
**Clinical year (4&5)**	1.57 (0.93-2.68)	0.094	1.21 (0.67-2.19)	0.524
**Tuberculosis case contact (yes)**	1.57 (1.11-2.21)	0.041	1.91 (1.01-3.61)	0.047
**History of extra clinical exposure**	2.78 (1.39-5.54)	0.004	3.39 (1.60-7.16)	0.001

OR = Odds Ratio.

When veterinary students were analysed separately, none of the factors studied were found to be significantly associated with TB.

No student was found with active TB disease and no serious reaction to TST like anaphylaxis or ulceration was observed.

## Discussion

The prevalence of TB infection was significantly higher among medical students than veterinary students. This suggests that medical students could be more exposed to TB infection than the veterinary students. The prevalence of TB infection among the medical students at Makerere was higher than that of medical students in a considered high TB incidence city of Rio de Janeiro state, Brazil in 2005 which was 6.9% [6]. The higher prevalence among students in Makerere University could be related to the higher national TB prevalence rate in Uganda. In 2011, WHO estimated the prevalence of TB in Uganda to be 193 per 100000 population and that of Brazil at only 47 per 100000 population [2]. However, the prevalence of TB infection among the medical students was lower than that found among the general health care workers in Kampala (57%) [4] but comparable to those in Trinidad and in India, yet higher than those in south Africa and in Mexico [10]. The lower prevalence of TB infection among medical students compared to the health care workers in general in Kampala is likely to be due the fact that the average health care worker has spent more time in contact with patients than the average medical student has.

When data from the two schools was combined, the independent predictors of TB infection were: male sex, residence at home, being a medical student and a positive history of contact with a known TB case. Similar to previous findings [4,5], males were more likely to have TB infection than females. This is probably due to their risk taking behaviour like disregard of protective measures and other social issues like smoking and spending time in crowded places which exposes them to TB infection rather than a genetic factor [11].

We had initially anticipated finding a higher TB prevalence among students residing at the university or private halls of residence since there is likely to be overcrowding in these residences. A possible explanation for the higher TB infection among students residing at home could be the higher prevalence of TB in Kampala city and the community where these students reside. This explanation is further supported by the finding of high TB infection prevalence among the veterinary school students (which is considered to be the basal prevalence) and yet veterinary students are less likely to be exposed to TB infection in training. In addition, the prevalence of TB infection was already high among students in their first year of study in either school. Indeed the strongest association was observed with “residence at home” which was even higher than being a medical student.

The medical students were 1.56 times more likely to have TB infection than the veterinary students possibly due to the additional exposure to TB infection from the patients the medical students encounter during their training on the wards and the out-patient departments. The veterinary students are likely to be exposed to the bovine type of TB from the study animals; however, the likelihood is very small since the main route of infection would be through ingestion of poorly prepared infected animal products like meat or milk. These students do not consume the products of their study animals. Secondly, the majority of the study animals are healthy, only brought in for study purposes.

The higher likelihood of TB infection among medical students was in contrast to what would be expected given the findings that students in the veterinary school were significantly older than medical students and had a higher proportion of male students. Male sex and higher age are both predictors of TB infection. This suggests that medical students are at a greater risk of TB infection possibly due to hospital exposure.

History of contact with a known TB case was significantly associated with TB infection. This finding has also been shown by previous studies [12,13]. Persons in close contact with a case of pulmonary TB disease stand a great chance of infection with TB. This risk is higher if it is smear positive TB disease; there is poor ventilation, longer duration of exposure and exposures during cough-inducing procedures.

The independent predictors of TB infection among the medical students when considered separately were: male sex, having been in a day school in high school, residing at home, history of contact with a known TB case and history of extra clinical exposure.

The reason for the association of TB infection with having been in a day school in high school could be similar to that of residing at home. Students in day schools spend considerably more time in the community – where TB rates are high – compared to those in boarding schools hence the higher likelihood of being infected with TB. History of extracurricular clinical exposure was associated with TB infection probably because the students who engage in these activities spend more time with patients than those who don’t hence greater exposure. A number of medical students voluntarily engage in extra clinical training during official school breaks. This is usually done to enhance their acquired clinical skills especially in district hospitals under the supervision of the relevant medical officers/consultants.

There seemed to be no single independent variable that was statistically associated with TB infection among the veterinary students when considered separately. This is likely to be due to the limited sample size. With a larger sample size, factors like sex and history of contact with a TB case are likely to be significant.

Our study had some limitations. We could not readily differentiate TST reactions due to other environmental mycobacteria especially *M.bovis* which could account for some positive TST more so among the veterinary students. This is only possible with other more expensive tests like interferon-gamma assays which were unaffordable in this study. We were unable to assess HIV serostatus due to budgetary limitations. In an HIV positive individual, a TST induration of 5 mm or more is considered positive. It is therefore likely that we underestimated the prevalence of TB infection. Though the clinical officers who performed the TST were trained by an experienced trainer working with Mulago hospital TB clinic, their concordance with the trainer was not assessed.

## Conclusions

Medical students in Makerere University had a higher prevalence of TB infection than their counterparts in veterinary school. However, most TB infections among both groups of students seem to have been acquired from the community probably due to high prevalence of TB in the city. The higher prevalence of TB infection among the medical students could be due to the additional exposure to patients with TB in the hospital during training. Measures to control TB infection in medical schools and in health-care facilities in general as recommended by WHO [14] need to be emphasized. A longitudinal study and a booster effect study is recommended to evaluate the conversion rate. This would be more informative regarding the risk of TB infection among the medical students.

## Competing interests

The authors declare that they have no competing interests.

## Authors’ contributions

HM conceived the study, developed and implemented the protocol, analyzed the data, interpreted the results and wrote the first draft of the manuscript. AK and DB closely supervised the protocol development and implementation and gave insight in the epidemiology of TB infection. SM and PM contributed to the protocol development, analysis and interpretation of the results. JNK and CK extensively reviewed the protocol and helped in the statistical analysis. All authors participated in reviewing and editing of various drafts of the manuscript and they all read and approved the final manuscript.
